# Predictors of Knowledge of Coronary Intervention in a Group of PCI Patients

**DOI:** 10.5539/gjhs.v8n6p187

**Published:** 2015-11-05

**Authors:** Abdul Haseeb, Muhammad Bilal, Mudassir Iqbal Dar, Mohammad Hussham Arshad, Raamish Bin Amir, Sahibzada Muhammad Hamid Hussain, Sharmeen Kamran Mian, Maheen Javed, Ayesha Sultan, Arham Amir Arfeen

**Affiliations:** 1 Dow University of Health Sciences, Karachi, Pakistan; 2 Civil Hospital Karachi, Pakistan; 3 Aga Khan University Hospital, Karachi, Pakistan; 4 Department of Biological Sciences, The Lyceum, Karachi, Pakistan; 5 Department of Biological Sciences, Karachi Grammar School, Karachi, Pakistan

**Keywords:** coronary artery disease, knowledge, risk factors, post management, percutaneous coronary intervention, Pakistan

## Abstract

**Objective:**

This study was performed to assess the knowledge of CAD risk factors and post management of coronary intervention among sample of population who were hospitalized for PCI.

**Methodology:**

A cross-sectional, descriptive survey was conducted in Cardiology ward of a tertiary care hospital from July 2013 to May 2015 on 600 patients. A structured questionnaire was used to interview the patients. In univariate analysis, t-tests were employed to assess association of knowledge of CAD risk factors with gender, education level and monthly household income.

**Results:**

The mean score of participants with no education was 4.42 and patients with education of bachelors or higher was 8.59 (p-value: 0.01). Similarly, the mean score for participants with monthly household income less than 5000 was 3.32 and participants with income higher than 50,000 had a score of 8.31 (p-value: 0.01). Furthermore, only 28% (N=168) claimed aerobic exercise as a key part of angioplasty recovery.

**Conclusions:**

Our results indicate the lack of good level of knowledge of risk factors for CAD and post management of coronary intervention among PCI patients of Pakistan. There is urgent need for targeted educational programs on national basis to reduce mortality associated with CAD in Pakistani population.

## 1. Introduction

Cardiovascular diseases are leading cause of death as they account for 15 million deaths every year in the world (World Health Organization [WHO], 1999). The Centers for Disease Control and Prevention (CDC) has reported that approximately 61 million people in the United States are suffering from cardiovascular disease (Centers for Disease Control and Prevention, 2012). It is further classified as a cardinal source of physical disability, specifically in rapidly increasing aged population. Moreover, people of Indo-Asian origin have one of the greatest vulnerability to coronary artery disease (CAD) in the world (Joshi et al., 2007), and it is therefore unsurprising that CAD has now become the major cause of death in the Indo-Pakistan subcontinent (Lopez et al., 2006). Epidemiological data tells us that the third world countries, such as Pakistan and India, account for many of the cases of cardiovascular diseases globally, with a surprising 75% of all moralities (WHO, 1999). The scenario is disastrous in Pakistan and other third world countries, as there is a drastic increase in number of patients with ischemic heart disease every year. The cardiac arrest independently contributes to 25% of death secondary to ischemic heart diseases in Pakistan (Rasheed, Habib, Dar, Saeed, Mehjabeen, & Jahan, 2014).

In Pakistan, the major leading factors associated with Coronary artery disease (CAD)/myocardial infarction (MI) includes tobacco use, ghee (clarified butter) intake, raised fasting glucose, high cholesterol, paternal history of CVD, low income, low levels of education, obesity, hypertension, male gender, age, sedentary habits (Ismail et al., 2004). The National Health Survey of Pakistan (NHSP) has reported that the prevalence of hypertension is 17.9% and that of diabetes is 10% (Khan & Mallick, 1992). Furthermore, the prevalence rates for obesity in an urban Pakistani population are 22% and 37% in males and females, respectively, while high blood cholesterol is reported to be in 13% of Pakistani adults (Khan & Mallick, 1992). Similarly, tobacco consumption has been documented in 29% of adult Pakistani men (Khan & Mallick, 1992). It is further claimed that people of South Asian descent have highest tendency of acquiring aforementioned risk factors of CAD. Therefore it is expected that worldwide CAD epidemic will be most marked in Pakistan.

Deterrence of CAD is the most constructive way of withstanding CAD epidemic in developing country like Pakistan. Awareness and knowledge of risk factors for CVD has been deduced to be a prerequisite for change in attitude and has been most commonly employed by prevention programs (Bani & Hashim, 1999). Knowledge and awareness along with other factors are key elements to influence ones’ decision making and to inculcate behavioral change (Ford & Jones, 1991), which further provides reminder for actions (Becker, Maiman, Kirscht, Haefner, & Drachman, 1977). Previous studies have indicated that educational programs were productive in improving knowledge and behavior among elderly population (Huang, Chen, Yu, Chen, & Lin, 2002). Therefore evaluating the knowledge of PCI patients can help to guide public health programs designed to reduce risk factors of CAD.

After a robust literature search, no study was obtained addressing the knowledge and awareness of CAD risk factors among patients undergoing PCI. However, recent studies reported poor knowledge conducted on general population especially in lower middle class urban population of Karachi (Dodani et al., 2004). Therefore, this study was performed to assess the knowledge of CAD risk factors and post management of coronary intervention among sample of population who were hospitalized for PCI. Assessment of their knowledge can help to initiate measures that can reduce morbidity and mortality occurring as result of CVD.

## 2. Methodology

A cross-sectional, descriptive survey was conducted in Cardiology ward of a tertiary care hospital from July 2013 to May 2015. Six hundred consecutive patients between ages of 30-80 years old irrespective of their socio economical and educational background, who were admitted to the cardiology ward for coronary angiography, were involved in the study. The admitted patients were suspected with ischemic heart disease clinically or identified by cardiac tests like ETT, ECHO, and Thallium stress test. All coronary angiograms were carried out and interpreted by well experienced cardiologists. The CAD disease was defined as >50% of luminal narrowing. The selected patients underwent elective percutaneous coronary intervention (PCI) for their diagnosed CAD. However, the patients presented with having significant history of angioplasty (PCI) in the past and coronary artery by-pass grafting (CABG) were excluded from the study to avoid biased opinions.

A structured questionnaire was designed in English based on components from previous studies (Almas, Hameed, & Sultan, 2008). It comprised of three parts which included demographic features of the patients, knowledge with regards to CAD and the last part consisted of questions to test knowledge with regards to post management of coronary intervention. The second part of the questionnaire consisted of 10 risk factors and was scored out of 10 points in all. Every correct answer for a question was awarded 1 point with the questionnaire consisting of a total of 10 questions. The questionnaire was pilot tested before this study was conducted. A trained medical researcher recruited the patients who were planned for PCI and fulfilled inclusion criteria. After explaining the purpose of the study and taking written informed consent, participants were interviewed in Urdu and the answers were translated in English to enter in the research questionnaire. Furthermore, the participants were also allowed to refuse to answer any particular question or withdraw from the study anytime. The study was approved by the ethical review board of Dow university of Health sciences and permission was obtained from Civil Hospital Karachi.

The collected data was entered on Microsoft Excel and later it was imported on SPSS (V.19) for data analysis. We calculated mean and standard deviation for quantitative variables like age. However, frequency and percentages were calculated for all categorical variables like gender etc. In univariate analysis, t-tests were employed to assess the association of knowledge of CAD risk factors with gender, education level and monthly household income. P-value of less than 0.05 was considered as significant to deduce valid conclusions.

## 3. Results

Our study comprised of 600 participants and the response rate was 100%. The mean age of participants was 57.4±15.7 years. Our study consisted of 375 (62.5%) males and 225 (37.5%) females. Forty percent (N=240) of the patients had no formal education whereas 24% (N=144) were labor by profession. Moreover, 17.9% (N=107) of sample size had monthly household earning in the range of 5000-20,000 (PKR). Thirty three percent (N=198) patients responded CVD for family history of any disease. Besides that, 27.5 % (N=165) were suffering from cardiac disease for greater than 15 years and 67% (N=402) of the participants were hypertensive. However, 25% (N=150) of the cardiac patients were cigarette smokers.

Fifty three percent (N=318) of patients believed that heart diseases were the primary cause of death in the Pakistani population, which was followed by cancer (20.0%), diabetes mellitus (9.0%), infection (8.0%), kidney diseases (7.0%) and stroke (1.0%). Forty five percent (N=270) thought that heart diseases are not preventable. Moreover, sixty five percent (N=390) of cardiac patients graded smoking as the greatest risk factor of CAD followed by family history of heart disease (61%), male sex (39%), HTN (31%) and increased cholesterol (28%). Alarmingly, 70% (N=420) considered that exercise does not prevent heart disease and 30% (N=180) participants rejected to quit smoking to prevent heart disease. Eleven percent (N=66) correctly defined angiography whereas 36% (N=216) had never heard this term and only 16% (N=96) claimed that angiography saves life of patient with heart attack.

A further statistical analysis was carried out and the mean scores were calculated. The mean score for CAD risk factors in our patients was 6.16±1.32 out of 10. In males the mean score was 5.98 whereas in females it was 6.20 (p-value: 0.32). The mean score for the patients who had received no primary education was 4.42 and for those patients with education of bachelors or higher the mean score was 8.59 (p-value: 0.01). Similarly, the mean score for participants with monthly household income less than 5000 was 3.32 and participants with income higher than 50,000 had a score of 8.31 (p-value: 0.01)

Furthermore, when patients were inquired about the knowledge with regards to post management of coronary intervention, only 28% (N=168) claimed aerobic exercise as a key part of angioplasty recovery. On average, 10.75% (N=65) of the patients were aware of the protocols and precautions associated with aerobic exercises. Fifty six percent (N=336) of the participants regarded cholesterol control as the major lifestyle change after stents, followed by BP control (53%), diabetes control (49%) and managing stress (39%). Forty one percent (N=246) considered reduction of salt intake as the major diet change after angioplasty. Moreover, patients responded MI (35%) and stroke (32%) as the major post angioplasty complication, where as high blood pressure was regarded as the major factor (41%) increasing the chances of restenosis.

**Table 1:** Depicts demographical characteristics of patients with CAD

Demographic characteristics	N	%
**Age (years) Mean± SD**	57.4±15.7	-
**Gender**		
Male	375	62.5
Female	225	37.5
**Level of education**		
Matriculation (grade 10)	145.2	24.2
Intermediate (grade 12)	81	13.5
Bachelors	61	10.1
Masters	72	12.0
No education	240	40.2
**Profession**		
Labor	144	24.0
Businessman	35	5.9
Government employee	47	7.9
Private employee	67	11.2
Housewife	150	25.0
Retired	156	26.0
**Monthly household income**		
Less than 5000	63	10.5
5000-20000	107	17.9
20001-50000	97	16.2
50001-100000	17	2.9
Greater than 100000	9	1.5
Not applicable	306	51.0
**Family history of any disease**		
Cardiovascular disease	198	33.0
Diabetes	66	11.0
Hyperlipidemia/Dyslipidemia	78	13.0
Cerebrovascular disease	48	8.0
Renal disease	108	18.0
None	102	17.0
**For how long have you been suffering from Cardiac disease**		
< 6 Months	66	11.0
6 Months - 2 Years	144	24.0
2.1 - 5 Years	108	18.0
5.1 - 15 Years	117	19.5
> 15 Years	165	27.5
**Smoking/Tobacco Use**		
Never used	432	72.0
Ex-user	18	3.0
Current user	150	25.0
**Clinical characteristics**		
Hypertension	402	67.0
Diabetes Mellitus	330	55.0

**Table 2:** Depicts knowledge of CAD risk factors and angiography

Variable	N	%
**Do you think that heart diseases are preventable?**		
Yes	210	35.0
No	270	45.0
Don’t know	120	20.0
**Are you aware of the following risk factors of Coronary artery disease**?		
Age	306	51.0
Male sex	234	39.0
High blood pressure	186	31.0
Family history of heart disease	366	61.0
Smoking	390	65.0
Increase Cholesterol	168	28.0
Diabetes	150	25.0
Sedentary life style	114	19.0
Obesity	162	27.0
Fatty food consumption	144	24.0
**Do you think that exercise prevents heart disease?**		
Yes	180	30.0
No	420	70.0
**Do you think you will consider quitting smoking to prevent heart disease?**		
Yes	420	70.0
No	180	30.0
**What is angioplasty?**		
Medicine for heart disease	60	10.0
Major operation	258	43.0
Never heard of it	216	36.0
Minor operation for treating heart disease	66	11.0
**Do you think that angioplasty/CABG saves lives of people who have heart attack?**		
Yes	96	16.0
No	144	24.0
Don’t know	360	60.0
**Do you think that Cardiac patients with heart disease have to take medications for life?**		
Yes	204	34.0
No	48	8.0
Don’t know	348	58.0

**Table 3:** Depicts knowledge regarding post management of coronary intervention

Variables	N	%
**Do you know that aerobic exercise is a key part of angioplasty recovery?**		
Yes	168	28.0
No	432	72.0
**If yes, are you aware with following precautions?**		
Wait 90 minutes after eating to exercise	66	11.0
Take 5 minutes to warm up before aerobic activity	54	9.0
Start slowly and increase your activity gradually	60	10.0
Don’t sit down immediately after exercise otherwise you will have heart palpitations	78	13.0
**Are you aware with following lifestyle changes after stents?**		
Reduction in calorie intake and maintain healthy weight	228	38.0
Stop smoking	216	36.0
BP control	318	53.0
Cholesterol control	336	56.0
Diabetes under control	294	49.0
Manage stress	234	39.0
**Are you aware with diet changes to be considered after angioplasty?**		
Increase fresh fruits and vegetables in daily diet	138	23.0
Increase consumption of sea food (Fish)	222	37.0
Reduction of caffeinated products and avoid all alcoholic beverages	186	31.0
Decrease sugar rich foods	228	38.0
Decrease salt intake	246	41.0
**Are you aware with post angioplasty complications?**		
Myocardial infarction (Heart attack)	210	35.0
Arrhythmia	162	27.0
Restenosis	114	19.0
Allergic reaction to the dye used to locate blockage	90	15.0
Emergency CABG	108	18.0
Stroke	192	32.0
Kidney damage	108	18.0
**Which of the following factors can increase the chances of restenosis?**		
Uncontrolled sugar level	180	30.0
Continued cigarette smoking	162	27.0
High blood pressure	246	41.0
High LDL levels	174	29.0

## 4. Discussion

In this study, majority of the cardiac participants (65.0%) correctly recognized Cigarette smoking as a risk factor for CAD. It is a proven fact that smoking tobacco increases chances of CAD by two folds (Almas, Hameed, & Sultan, 2008). The results are consistent with a study conducted on university students in Pakistan (Price, Mowbray, Lee, Rumley, Lowe, & Fowkes, 1999). However, a cross sectional study carried out in 4 tertiary care hospitals revealed that only 31% of sample population was aware about the fact (Choudhury & Marsh, 1999). The knowledge about smoking as risk factor in our sample population is plentiful. Despite of the fact, 30% rejected to quit their habit as a precautionary measure for CAD. This signifies the fact that prompt actions must be taken by the Health State department in forms of anti tobacco media coverage in order to prevent our cardiac patients from smoking. According to work of Hu FB et al, recommendations by consultants to stop smoking along with intervention managed by nurses have yielded great outcomes (Hu et al., 2000). Epidemiological data has revealed that decrease in smoking caused a drop of 13% age wise incidence of CAD (Niebauer et al., 1997). Moreover, the ex-tobacco users were less aware with risk factors of CAD as their mean knowledge score was lower than those who never used it. The results are identical with deductions of studies conducted in the US and Scotland (Ford & Jones, 1991). The results could be attributed to the fact that tobacco users are less prepared to appreciate the adverse effects of tobacco on their health as compare to the non-users (Woodward, Bolton-Smith, & Tunstall-Pedoe, 1994).

Surprisingly, in our study only 19% considered sedentary life style as a risk factor of CAD and 70% responded that exercise has no role in preventing heart disease. The results are consistent with a study conducted in US (Zerwic, King, & Wlasowicz, 1997). According to the American Heart Association physical inactivity has been declared as a major modifiable risk factor for cardiac diseases (Fletcher et al., 1992). An exercise routine of six daily 10 minute sessions with a bicycle ergometer at an intensity of 802% maximal heart rate should be recommended to the cardiac patients as it is closely linked with improvements in endothelial dependent vasodilatation of coronary arteries after four weeks (Bayne-Smith et al., 2004).

Furthermore, knowledge regarding modifiable risk factors like HTN, high Cholesterol level and DM was inadequate and only 30% of the people considered it as a risk factor for cardiac disease. The results are in agreement with Jaffer et al where participants from South Asian population also had insufficient knowledge regarding these risk factors (Ismail et al., 2004). However, our results are contradictory to the findings of the study conducted by students of Warsaw University (Belardinelli, Georgiou, Cianci, & Purcaro, 1999). Since these modifiable risk factors are key elements in prevention of CAD, it is an utmost necessity to educate cardiac patients regarding them. A physician must emphasize on importance of controlling these risk factors to susceptible patients and mass media campaign must be carried out to increase awareness regarding risk factors of heart disease.

Majority of the participants who were planned for angioplasty were unable to correctly define the procedure. Alarmingly, 36% of the participants never heard about the procedure before. This highlights that cardiac patients are not well aware with treatment modalities of acute myocardial infarction, which leads to development of false illusions. Majority of the participants declaimed that the cost of the treatment for angina was the major cause of their delayed treatments, followed by a lack in expertise and surprisingly also due to the fear of angiography. The similar reasons have limited use of angiography and revascularization internationally as well. This is due to the fact that widespread expertise in these technical procedures is also absent in Western countries (Selby et al., 1996). Moreover, it is also proven that risk of adverse Cardiac events in CAD patients is much lesser among hospitalized patients who undergo angiography (Selby et al., 1996). Due to this evidence we can suggest that the patients with acute MI should be made more aware of the opportune use of these procedures.

Furthermore, our study revealed inadequate knowledge regarding the self-care practices involved in the post management of coronary intervention. It was evident that very few cardiac patients were aware of the fact that aerobic exercise is a cornerstone of angioplasty recovery. Majority of the participants were not knowledgeable about the protocols (http://www.everydayhealth.com/health-report/angioplasty-recovery-guide/staying-fit.aspx) associated with aerobic exercises. Therefore we suggest that physical therapists must be appointed and patients must be given mandatory training on the physical activities before their discharge from hospital. Patients must also be counseled on lifestyle changes that are supposed to be implemented after angioplasty as most of the patients did not consider smoking cessation, reduction in calorie intake and management of stress to be significant. A heart healthy diet aids the recovery process after an angioplasty, or any kind of heart disease related surgery (Belardinelli et al., 1999). A significant proportion of the participants were not aware about heart healthy diet. As a result, physicians must design a diet chart considering diet habits of the patient and the changes need to be made according to international guidelines in order to obtain a faster recovery after angioplasty (Belardinelli et al., 2014). The awareness regarding post angioplasty complications and factors that increases chance of restenosis will increase patients’ adherence to medications and will also reduce morbidity and mortality rates among cardiac patients.

To the best of our knowledge, this is the first study of its kind where knowledge of risk factors of CAD and post management of coronary intervention was comprehensively assessed on a large sample population. However, there are few limitations in the study. Firstly, the study was single city in nature as it was only conducted in Karachi and majority of participants belong to low socioeconomic status and illiterate population. Due to these limitations the scope of this study could not cover all of the population of Pakistan and is only bound to a small fraction of it. Since the level of knowledge was assessed using a structured questionnaire, participants might have responded in favor of all risk factors mentioned in the questionnaire. As a result we might have overestimated the total level of knowledge in this population. Besides, relying on ability to recall answers of knowledge questions induces recall bias and we might have underestimated the knowledge in this population.

## 5. Conclusion

Our results indicate a striking lack of knowledge of modifiable risk factors and awareness regarding post management of coronary intervention among patients planned for PCI. Only 19% of our subjects correctly identified all the risk factors of CAD, and a mere of 25% had a good knowledge regarding post management. Moreover, participants considered smoking as a major risk factor for CAD, but in spite of this very few agreed to quit smoking. The outcomes of our study could be of great help to higher authorities as we have identified subset of population that specially needs to be targeted through awareness programs. The results suggest that urgent and targeted awareness programs about the relationship of obesity and exercise with CAD must be initiated. Our results also call for actions to be taken with the help of local government and nongovernmental organizations to increase level of knowledge specifically among cardiac patients. The awareness programs should target attitudes, perceptions and capabilities of cardiac patients. Physicians should also educate their patients as patients in our society usually rely on doctors for first hand information. More studies with multicentre cluster sampling must be conducted to evaluate the knowledge of PCI patients regarding risk factors and post management self-care practices.

**Abbreviations**
CAD: Coronary Artery Disease
CVD: Coronary Vascular Disease
HTN: Hypertension
BP: Blood Pressure
MI: Myocardial Infarction
CDC: Centers for Disease Control and Prevention
NHSP: National Health Survey of Pakistan
